# Retraction: Analysis of *Mycobacterium tuberculosis*-Specific CD8 T-Cells in Patients with Active Tuberculosis and in Individuals with Latent Infection

**DOI:** 10.1371/journal.pone.0223028

**Published:** 2019-09-19

**Authors:** The PLOS ONE Editors

Following publication of this article [1], concerns were raised about the flow cytometry dot plot images in Fig 1B, and these concerns were brought to the attention of the *PLOS ONE* Editors by the authors.

The following concerns were identified:

- LTBI 16kDa and Rv1490 panels show regions of similarity;
- T0 16kDa and Rv1490 panels show regions of similarity;
- T4 Ag85, ESAT-6, and Hsp65 panels show regions of similarity;
- T4 Nil, 16kDa, Rv1490, and Rv1614 panels show regions of similarity.

The authors stated that the Service Facility at the University Hospital in Palermo performed all FACS analyses and provided printed FACS plots. The data were collected in 2006/7 and the original raw flow cytometry data (fcs) files are no longer available.

Owing to the unavailability of the raw data, the above issues could not be resolved. In light of the concerns regarding the preparation of Fig 1B and the handling of flow cytometry data, the *PLOS ONE* Editors retract this article.

JN, THMO agreed with the retraction. AM, MRK did not respond. NC, Giuliana Guggino, SM, PDC, MLB, DG, Alessandro Sanduzzi, FD did not agree with retraction. Giuseppe Gelsomino, LT, Alfredo Salerno could not be reached.
